# Supplementary material for: Sustained-input switches for transcription factors and microRNAs are central building blocks of eukaryotic gene circuits
Source: Genome Biol. 2013 Aug 23;14(8):R85. doi: 10.1186/gb-2013-14-8-r85 (PMC4054853; doi:10.1186/gb-2013-14-8-r85)
Supplement: Additional file 5 — HTML Browsable Motif Output. Zipped folder containing all WaRSwap and FANMOD motif output, viewable in a web browser. [file gb-2013-14-8-r85-S5.ZIP › HTML_browsable_motif_output/FANMOD_ath_tair9/sigs_fanmodm-2000.pvals.heatmaps.html/motif_id_46_011101002_tftype_ath_upstream_-3000_0.html]

```
BG_MODEL = FANMOD
MOTIF_ID = 46_011101002
TF_TYPE = ath
UPSTREAM = -3000_0


PVals
FN_0.2	FN_0.4	FN_0.6	FN_0.8
dg_60.genes	0.052	0.001	0.001	0.831
dg_70.genes	0.051	0.004	0	0.837
dg_80.genes	0.054	0.004	0	0.844

ZScores
FN_0.2	FN_0.4	FN_0.6	FN_0.8
dg_60.genes	1.68	2.676	3.333	-1.195
dg_70.genes	1.681	2.633	3.45	-1.254
dg_80.genes	1.635	2.685	3.449	-1.237

StDevs
FN_0.2	FN_0.4	FN_0.6	FN_0.8
dg_60.genes	22604.593	25654.283	7838.726	1063.014
dg_70.genes	22229.562	25523.254	7630.651	1071.092
dg_80.genes	22988.953	25761.822	7594.321	1077.79
```
